# Characteristics of visits and predictors of admission from a paediatric emergency room in Saudi Arabia

**DOI:** 10.1186/s12873-021-00467-7

**Published:** 2021-06-21

**Authors:** Mohammad H. Al-Qahtani, Abdullah A. Yousef, Bassam H. Awary, Waleed H. Albuali, Mohammed A. Al Ghamdi, Reem S. AlOmar, Nouf A. AlShamlan, Haneen A. Yousef, Sameerah Motabgani, Naheel A. AlAmer, Kawthar M. Alsawad, Fatimah Y. Altaweel, Kawther S. Altaweel, Roaya A. AlQunais, Fatima A. Alsubaie, Malak A. Al Shammari

**Affiliations:** 1 Department of Paediatrics, Imam Abdulrahman Bin Faisal University, College of Medicine, Dammam, Saudi Arabia; 2 King Fahd Hospital of the University, Al-Khobar, Saudi Arabia; 3 Department of Family and Community Medicine, Imam Abdulrahman Bin University, Dammam, Saudi Arabia; 4 College of Medicine, Imam Abdulrahman bin Faisal University, Dammam, Saudi Arabia

**Keywords:** Paediatric, Emergency room, Admissions, Saudi Arabia

## Abstract

**Background:**

The Emergency Room (ER) is one of the most used areas in healthcare institutions. Problems with over utilisation and overcrowding have been reported worldwide. This study aims at examining the characteristics of paediatric ER visits, the rate of hospital admissions and its associated predictors at King Fahd Hospital of the University in the Eastern Province of Saudi Arabia.

**Methods:**

This is a retrospective, medical record-based study. Variables included gender, age group, nationality, complaints, Triage level, shifts and seasons. Descriptive statistics were reported as frequencies/percentages. *P*-values were obtained through a Chi-Squared test while unadjusted and adjusted odds ratios were estimated by binary logistic regression, where admission was considered as the outcome.

**Results:**

The total number of paediatric patients included was 46,374, and only 2.5% were admitted. Males comprised 55.4% while females comprised 44.6%. The most common age group were toddlers, and 92.4% of the total sample were Saudis. The most common complaint was fever (26.9%) followed by respiratory symptoms (24.9%). Only 7 patients (0.02%) were classified as triage I (Resuscitation), and most were triage IV (Less urgent) (71.0%). Most visits occurred during the winter months. Adjusted ORs showed that neonates had higher odds of admission (OR = 3.85, 95%CI = 2.57–5.76). Moreover, those presenting with haematological conditions showed an OR of 65.49 (95%CI = 47.85–89.64), followed by endocrine conditions showing an OR of 34.89 (95%CI = 23.65–51.47). Triage I had a very high odds of admission (OR = 19.02, 95%CI = 2.70–133.76), whereas triage V was associated with a very low odds of admission (OR = 0.30, 95%CI = 0.23–0.38).

**Conclusions:**

A low rate of hospital admission was found in comparison with other rates worldwide. This was mostly attributed to an alarmingly high number of non-urgent ER visits. This further emphasises the problem with improper use of ER services, as these cases should be more appropriately directed towards primary healthcare centres. Further studies to examine the impact of prioritising patients in the ER based on the identified predictors of hospital admission, in addition to the standard triage system, are suggested.

## Background

The Emergency Room (ER) remains one of the most utilised areas for paediatric patients in healthcare institutions. It continues to suffer from prolonged length of stay, overcrowding and extended waiting hours, all of which may lead to adverse events [1]. This is especially the case in Saudi Arabia (SA), where the ER may still be considered the main point of entrance to the healthcare system [2].

Paediatric ER visits vary worldwide. For example, in the US, it comprised an estimated 20% of all ER visits. In SA, children were reported to comprise 32.6% of all ER visits in a tertiary hospital in the Capital city of Riyadh [3].

The Canadian Triage and Acuity Scale (CTAS) has been implemented in almost all tertiary care hospitals in SA. The scale is used to manage and prioritise patients according to the severity of their condition and is based on five levels. It ensures that patients who are critically ill or injured are attended to before the less ill or injured patients [4]. The implementation of this system highlighted the significant problem in which patients arrive at the ER with non-urgent and mild conditions where they would have been more appropriately seen by a primary care physician.

According to recent research of one tertiary hospital in SA, 78.5% of visits to the ER were of non-urgent cases [5]. Another research which looked into three tertiary hospitals in SA found that non-urgent cases comprised 53% of all ER visits and examining paediatric ER visits specifically, non-urgent cases ranged between 35.9 and 59.5% [6, 7].

This problem has been a focus of the government lead by the Ministry of Health, in which an attempt has been made to reform the healthcare system by including primary healthcare services in its reform strategy for 2010–2020. The strategy aimed at increasing the number and quality of primary care centres including its preventive services. Additionally, more programmes and initiatives for the Saudi Vision 2030 focus on public health and primary healthcare services [8].

This study aims at assessing the characteristics of paediatric ER visits, as well as estimating the rate of admissions and its predictors in one large teaching hospital in the Eastern Province of SA.

## Methods

### Study setting and design

Following ethical approval, we conducted this retrospective study at King Fahd Hospital of the Imam Abdulrahman Bin Faisal University in the Eastern Province of SA. The Paediatrics’ ER contains 7 observation beds, 2 examination rooms, 1 triage room, a waiting area, and a minor surgical room. Major trauma cases are treated in the resuscitation room (for both adults and paediatrics). Paediatric ER is supported by pharmacy, radiology services, and the ability to refer the patient for admission as needed. The ER staff is made up of six qualified paediatric emergency consultants, as well as eight paediatric specialists devoted to emergency coverage. This is in addition to rotating paediatric program residents in different levels, one each month. The initial triage in the registration area is done by a qualified nurse while the paediatric ER triage is done by the assigned paediatric nurse and the rotating residents. The ER staff work in three shifts to cover the 24 h, these are the morning shift (8:00 am-16:00 pm), the evening shift (pm 16:00–00:00 am), and the night shift (am 00:00–8:00 am). To cover the needs in the morning and night shifts, there are two to four physicians, and two to four nurses except for the evening shift where there is a minimum of three physicians. Some shifts also involve rotating interns and students for training and teaching.

### Study population

The study included all patients presented to the paediatric ER, aged from 1 day to < 14 years old between the 1st of January 2018 and the 31st of December 2018. According to the hospital’s policy, the paediatric department is responsible for patients who fall within this age range.

### Data collection

A structured data collection sheet to collect data from the patient’s electronic medical records was used. Data included information about patient’s age, sex, season in which the patient presented in, chief complaint – as reported by the caregiver and categorised by the involved system, which shift received the patient, and whether admission to the hospital ward was ordered. These variables were chosen based on the study objectives as well as the literature and availability of the data.

### Statistical analysis

The age groups were allocated as the following: neonates (< 29 days), infants (29 days–1 year), toddlers (> 1 year–3 years), pre-schoolers (> 3 years–6 years), schoolers (> 6 years–12 years) and adolescents (> 12 years–14 years). Nationality as: Saudis and non-Saudis. The time of visits was grouped based on the season; Winter (December, January, February), Spring (March, April, May), Summer (June, July, August), and Autumn (September, October, November). Shifts were divided into three groups: morning (8:00 am-16:00 pm), evening shift (pm 16:00–00:00 am), and night shift (am 00:00–8:00 am). The chief complaints were categorized according to the system involved. The triage categories were according to the CTAS implemented in the hospital, in which I is for resuscitation, II for emergent, III for urgent, IV for less urgent, and V for non-urgent [4]. Descriptive statistics were presented as frequencies and percentages. The main outcome was whether the patient was admitted to hospital or not. The Chi-squared test was performed to compute the *P*-values, and statistical significance was set at *P* < 0.05. The Bonferroni adjustment was used to adjust for multiple comparisons. Unadjusted and adjusted Odds ratios (ORs) were drawn through multivariable binary logistic regression, where the reference category was the most frequent. The final model was chosen based on appropriate model diagnostics. Statistical analysis was performed in Stata Software 15 Software [9].

## Results

The total number of ER visits by paediatric patients was 46,374. The sociodemographic characteristics of patients are presented in Table 1. The sample consisted of 55.4% males and 44.6% females. The highest number of visits belonged to the toddlers age group (32.0%), followed by schoolers (24.9%). The smallest number of visits were for the neonates (0.9%). Most patients were of the Saudi nationality.

**Table 1:** Sociodemographic characteristics of paediatric patients 2018

Characteristic	N (%) 46,374 (100)
*Sex*	
Males	25,675 (55.4)
Females	20,699 (44.6)
*Age group*	
Neonates (> 29 days)	408 (00.9)
Infants (29 > 11 months)	6813 (14.7)
Toddler (1–3 years)	14,833 (32.0)
Pre-schooler (4–6 years)	9498 (20.5)
Schooler (7–12 years)	11,580 (24.9)
Adolescent (13–14 years)	3242 (07.0)
*Nationality*	
Saudi	42,825 (92.4)
Non-Saudi	3549 (07.6)

The ER characteristics of paediatric patients are presented in Table 2. Of the total sample, only 2.5% of patients were admitted. The highest number of complaints recorded was for fever followed by respiratory symptoms (27.0 and 24.9% respectively). With regards to triage, only 0.02% were for triage I and 0.02% for triage II, whereas triage IV and V made up 71.0 and 26.2% of the visits. Most visits occurred in the evening shift and the winter season had the highest rate of visits (31.9%).

**Table 2:** ER visit characteristics of paediatric patients during 2018

Characteristic	N (%) 46,374 (100)
*Admission*	
Yes	1134 (02.5)
No	45,240 (97.5)
*Complaints*	
Cardiology	75 (00.2)
Dental	231 (00.5)
Dermatology	1751 (03.8)
Endocrine	202 (00.4)
ENT	2975 (06.4)
Fever	12,492 (27.0)
Genitourinary	377 (00.8)
GI	7078 (15.3)
Haematology	327 (00.7)
Ophthalmology	1028 (02.2)
Musculoskeletal	499 (00.9)
Neurology	462 (01.0)
Respiratory	11,554 (24.9)
Surgery	377 (00.8)
Toxicology	387 (00.8)
Trauma	5212 (11.2)
Others	1447 (03.1)
*Triage*	
I	7 (0.02)
II	10 (0.02)
III	1242 (02.6)
IV	32,931 (71.0)
V	12,184 (26.2)
*Shift*	
Morning	13,649 (29.4)
Evening	21,568 (46.5)
Night	11,157 (24.1)
*Season*	
Winter (December, January, February)	14,800 (31.9)
Spring (March, April, May)	11,563 (25.0)
Autumn (September, October, November)	11,970 (25.8)
Summer (June, July, August)	8041 (17.3)

When exploring the association between sociodemographic characteristics and admissions, the age group variable was found to be highly statistically associated with admissions (*P* < 0.001). Analysing this further, comparisons between all age groups were statistically significant, except for adolescents with other age groups. Further, the rate of admission was not found to be different between males and females (Table 3).

**Table 3:** Associations between admissions and sociodemographic characteristics of paediatric ER patients during 2018

Characteristic	Admission		*P*-value
	No 45,240 (97.55)	Yes 1134 (02.45)	
*Sex*			0.91
Males	25,049 (97.6)	626 (02.4)	
Females	20,191 (97.5)	508 (02.5)	
*Age group*			< 0.001
Neonates (> 29 days)	371 (90.9)	37 (09.1)	
Infants (29 > 11 months)	6572 (96.5)	241 (03.5)	
Toddler (1–3 years)	14,536 (98.0)	297 (02.0)	
Pre-schooler (4–6 years)	9334 (98.3)	164 (01.7)	
Schooler (7–12 years)	11,265 (97.3)	315 (02.7)	
Adolescent (13–14 years)	3162 (97.5)	80 (02.5)	
*Nationality*			0.44
Saudi	41,771 (97.5)	1054 (02.5)	
Non-Saudi	3469 (97.7)	80 (02.3)	

Associations between ER visit characteristics and admission are presented in Table 4. The presenting complaints, triage and shifts were found to be highly statistically associated with admissions at the *P* < 0.001 level. The Endocrine and Haematological complaints, as well as triage group V, were found to have contributed mostly to the high statistically significant *p*-value when compared to other groups following multiple comparison testing. For shifts, the only statistical difference was found for the comparison between the evening and the morning shift (Bonferroni adjusted *p*-value < 0.001). No significant association was observed for the season.

**Table 4:** Associations between admissions and ER visit characteristics of paediatric ER patients during 2018

ER visit characteristics	Admission		*P*-value
	No 45,240 (97.55)	Yes 1134 (02.45)	
*Complaint*			< 0.001
Cardiology	71 (94.7)	4 (05.3)	
Dental	231 (100.0)	0	
Dermatology	1735 (99.1)	16 (00.9)	
Endocrine	133 (65.8)	69 (34.2)	
ENT	2950 (99.2)	25 (00.8)	
Fever	12,375 (99.1)	117 (00.9)	
Genitourinary	356 (94.4)	21 (05.6)	
GI	6920 (97.7)	158 (02.3)	
Haematology	193 (59.0)	134 (41.0)	
Ophthalmology	1021 (99.3)	7 (00.7)	
Musculoskeletal	395 (99.0)	4 (01.0)	
Neurology	422 (91.3)	40 (08.7)	
Respiratory	11,250 (97.4)	304 (02.6)	
Surgery	360 (95.5)	17 (04.5)	
Toxicology	358 (92.5)	29 (07.5)	
Trauma	5085 (97.6)	127 (02.4)	
Others	1385 (95.7)	62 (04.3)	
*Triage*			< 0.001
I	3 (42.9)	4 (57.1)	
II	7 (70.0)	3 (30.0)	
III	1063 (85.6)	179 (14.4)	
IV	32,068 (97.4)	863 (02.6)	
V	12,099 (99.3)	85 (00.7)	
*Shift*			< 0.001
Morning	13,263 (97.2)	386 (02.8)	
Evening	21,099 (97.8)	469 (02.2)	
Night	10,878 (97.5)	279 (02.5)	
*Season*			0.93
Winter (Dec, Jan, Feb)	14,432 (97.5)	368 (02.5)	
Spring (Mar, Apr, May)	8266 (97.6)	199 (02.4)	
Autumn (Sep, Oct, Nov)	11,679 (97.6)	291 (02.4)	
Summer (Jun, Jul, Aug)	7846 (97.6)	195(02.4)	

The unadjusted and adjusted ORs of admissions in relation to the sociodemographic and ER visit characteristics are presented in Table 5. The analysis shows that before and after adjusting for other variables, there is higher odds of admission for both neonates and infants, although the risk is almost fourfold for neonates (Adjusted OR = 3.85, 95%CI = 2.57–5.76 and Adjusted OR = 1.89, 95%CI = 1.56–2.28 respectively). With regards to the complaint, the highest odds of admission were for haematological illnesses both before and after adjustment, followed by endocrinal conditions when compared to fever which is statistically significant. In comparison with triage IV, triage I had a 19.02 OR for admission, followed by triage II (OR = 10.66, 95%CI = 1.98–57.23). Triage V had the lowest odds of admission both before and after adjustment and was statistically significant (Adjusted OR = 0.30, 95%CI = 0.23–0.38). Morning shifts were associated with a relatively high odd of admission, although they were not significant after adjustment, and neither were the seasons of the visit.

**Table 5:** Unadjusted/adjusted ORs of admissions according to sociodemographic/ER visit characteristics of paediatric patients during 2018

Characteristics	Risk of admission	
	Unadjusted OR (95%CI)	Adjusted OR (95% CI)
*Sex*		
Males	Reference	
Females	1.00 (0.89–1.13)	1.06 (0.93–1.20)
*Age group*		
Neonates (> 29 days)	4.88 (3.41–6.97)	3.85 (2.57–5.76)
Infants (29 > 11 months)	1.79 (1.51–2.13)	1.89 (1.56–2.28)
Toddler (1–3 years)	Reference	
Pre-schooler (4–6 years)	0.85 (0.70–1.04)	0.91 (0.74–1.13)
Schooler (7–12 years)	1.36 (1.16–1.60)	1.09 (0.90–1.31)
Adolescent (13–14 years)	1.23 (0.96–1.58)	0.84 (0.63–1.12)
*Complaint*^a^		
Cardiology	5.95 (2.14–1.64)	2.61 (0.75–9.11)
Dermatology	0.97 (0.57–1.64)	1.16 (0.68–1.98)
Endocrine	54.87 (38.93–77.33)	34.89 (23.65–51.47)
ENT	0.89 (0.58–1.38)	1.15 (0.71–1.58)
Fever	Reference	
Genitourinary	6.23 (3.87–10.04)	6.66 (4.08–10.86)
GI	2.41 (1.89–3.07)	2.06 (1.60–2.66)
Haematology	73.43 (55.17–97.73)	65.49 (47.85–89.64)
Ophthalmology	0.72 (0.33–1.55)	0.86 (−.39–1.86)
Musculoskeletal	1.07 (0.39–2.91)	1.04 (0.32–3.32)
Neurology	10.02 (6.91–14.54)	6.19 (4.07–9.43)
Respiratory	2.85 (2.30–3.54)	2.65 (2.12–3.32)
Surgery	4.99 (2.97–8.39)	5.42 (3.14–9.35)
Toxicology	8.56 (5.62–13.03)	4.50 (2.82–7.15)
Trauma	2.64 (2.05–3.40)	2.30 (1.75–3.02)
Others	4.73 (3.46–6.47)	4.78 (3.38–6.76)
*Triage*		
I	49.54 (11.07–221.70)	19.02 (2.70–133.76)
II	15.92 (4.11–61.98)	10.66 (1.98–57.23)
III	6.25 (5.26–7.43)	4.08 (3.30–5.04)
IV	Reference	
V	0.26 (0.20–0.32)	0.30 (0.23–0.38)
*Shift*		
Morning	1.30 (1.14–1.50)	1.14 (0.98–1.32)
Evening	Reference	
Night	1.15 (0.99–1.34)	1.10 (0.93–1.30)
*Season*		
Winter (Dec, Jan, Feb)	Reference	
Spring (Mar, Apr, May)	0.94 (0.79–1.12)	0.83 (0.69–0.99)
Autumn (Sep, Oct, Nov)	0.97 (0.83–1.14)	0.94 (0.79–1.10)
Summer (Jun, Jul, Aug)	0.97 (0.81–1.16)	0.84 (0.69–1.01)

^a^Dental category dropped in regression analyses due to zero counts

**Fig. 1:**
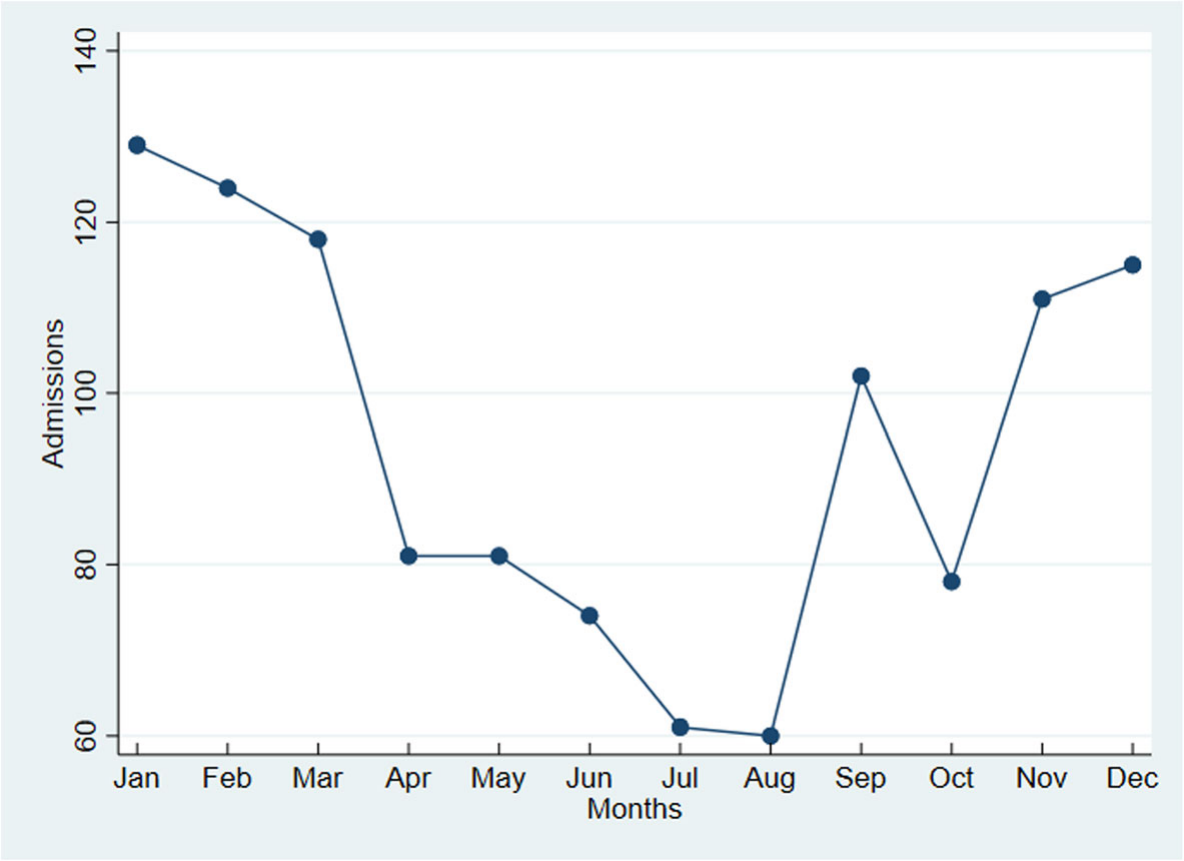
Number of admissions from ER paediatric visits during 2018

Figure 1 shows the number of admissions by month of visits. It can be seen that the lowest numbers of admission were during July and August, whereas the highest were for January, February and March.

## Discussion

This study supports the evidence that paediatric ERs are very much being used by patients as the first point of contact with the healthcare system, rather than visiting their local primary health care physician. This is a problem which should have eased with the current governmental initiative in promoting the use of primary healthcare.

### Characteristics of paediatric ER visits

Within this data, it was found that the most common paediatric age group was for toddlers aged between 1 < 3 years. Similar distributions have been found in other paediatric ERs in the US [10], Lebanon [11], and in another Saudi hospital located at the capital city of Riyadh [12]. Toddlerhood is a critical period where children start crawling, walking, learning to run, climbing, and jumping. Therefore, they may be prone to accidents or minor injuries. Moreover, the least common age group in our data were neonates, which is in sharp contrast to data from Pakistan which found that neonates had the highest rate of visits [13]. Cultural differences may have played a role where mothers attempt to treat theirs infants with herbal medicines or over the counter medications when they display certain symptoms such as infantile colic rather than presenting their child to a doctor [14].

Fever was the highest presenting complaint in our data. This is similar to other studies in Thailand and Lebanon [11, 15]. Fever is a general complaint and may be a sign of a wide spectrum of illnesses which may explain how it became the most common presenting complaint. Like the Thailand study [15], respiratory symptoms were the second most common complaint, which usually presents in the form of coughing, sneezing and difficulty in breathing. Such symptoms may be brought by air pollution, second-hand smoking or more strongly by changes in weather. Indeed, our findings show that weather has been found to play a role in the rates of ER visits. The highest number of visits were found to be during the winter, in November and December, but more specifically in January. Higher winter visits are mainly due to the common viral infections occurring in this period such as bronchitis, viral upper respiratory tract infections as well as complicated viral infections superimposed with bacterial infections. On the other hand, the summer season had the lowest number of visits. Prior to the COVID-19 epidemic, families tend to travel abroad during this season [16]. Other studies have also found similar patterns to that of our analysis both locally and internationally [12, 15]. Two Saudi studies, one based in Riyadh (1997) and the other in the Eastern region (2007), have found that non-urgent cases belonging to triage IV and V comprised between 35.9 and 59.5% of ER visits [6, 7]. Although, these two studies have been published a long time ago, the issue remains persistent and is worsening. Approximately two thirds of our patients were classified in Triage category IV, indicating they are non-urgent cases which may have been better dealt with in a primary care setting. To date, parents and patients in general in SA still prefer to seek medical services in the ER. Possible explanations may have to do with a lack of knowledge of a primary care centre near their home, long appointment hours in the primary care centre, dissatisfaction with the primary care physician, accessibility issues or even a perception that the condition is urgent and warrants medical attention straight away [5, 17, 18]. The management of such non-urgent cases in the ER increases the burden on the ER staff which may subsequently affect the quality of care that should be reserved for the high priority cases.

### Admissions and their associated factors

The admission rate of paediatric ER cases in this study was 2.45%. It is similar to the 3.3% rate of admission reported in the US [19], but much smaller than that reported in Lebanon, South Korea, and Pakistan, where the rates were reported to be 9.8, 14.3 and 45% respectively [11, 13, 20]. A systematic review by Obermeyer et al. (2015) assessed emergency care in low and middle-income countries and found that the median paediatric admission rate was 22% [21]. Comparing it with the admission rate from this study further emphasizes the role of primary care centres in accommodating the vast majority of patients presenting in the ER. Similar to the South Korean study [20], we found no significant differences in admission rates between males and females.

Unlike studies conducted in the US, Lebanon, Pakistan and Egypt, analyses here have shown that the neonate age group had the highest odds of admission, even though they were the smallest number with regards to ER visits [11, 13, 22, 23]. Differences in rates may be attributed to the different disease prevalence in these countries which may be affected by genetics, accessibility and the type of healthcare provided in hospitals. A further explanation to the higher odds of admission of neonates could be related to the hospital regulations and protocols to rule out neonatal sepsis [24].

Haematological conditions also had the highest odds of admissions. The Saudi population, especially those residing in the Eastern Province, which this data is derived from, are known to have a high prevalence of sickle cell disease (SCD) in children [25]. The prevalence of genetic haematological diseases in SA may be lowered by educating new prospective couples and enforcing recommendations by premarital counselling, as they are yet to be obligatory. Our data contrasts with that of Pakistan and Nigeria, where respiratory complaints had the highest rate of admissions [13, 26]. In Lebanon, complaints related to cardiology were the highest [11].

Furthermore, Triage I was associated with the highest odds of admission in these patients. Similar patterns were found in a US study, although the Emergency Severity Index level was used rather than CTAS [27]. Triage V was associated with lower odds of admission, which again highlights that the ER may suffer from overcrowding due to the presentation of non-urgent cases.

Neither the shift nor the season of the paediatric patients visiting the ER had any association with the rates of admission. It may be of notice that Spring, Autumn and Summer, despite their non-statistical significance, did show lower odds of admission when compared to Winter. In Australia, high and low temperatures were significantly associated with an increase in paediatric ER admissions [28], this was also noted in the UK [29]. Higher admission rates in low temperatures may be explained by children being more prone to cold weather viruses that could subsequently cause bronchiolitis in under 2 years olds and ultimately lead to an increased ER admission [30].

Several limitations with this study have been identified. The study is based on retrospective medical records drawn from a single healthcare institution, which may compromise the generalisability of the results. Also, despite the large number of data, some confidence estimates were wide which may be due to the extremely small number of cases within cells.

## Conclusion

This study examined paediatric ER visits at a large teaching hospital in the Eastern Province of SA. It showed that the admission rate of ER visits is extremely low. It also showed that neonates, patients with haematological conditions and patients classified as triage I had the highest odds of admission. However, patients classified as triage V had lower odds of admission. Clear guidance should be used to inform patients and parents of the proper process for seeking medical assistance, as well as ensuring more accessibility to primary health care centres. Moreover, further analysis focused on the predictors identified in this research is highly recommended to inform hospital administrators of potential minor changes that could lead to significant reduction in the over utilisation of the ER.

## Data Availability

The data used in this study were obtained from the electronic medical records of patients and are not publicly available. However, a request may be put to Paediatric Department Head Dr. Abdullah A Yousef who is a co-author in this study.

## Abbreviations

ER: Emergency room
SA: Saudi Arabia
CTAS: Canadian triage and acuity scale
