# Correction to: Characteristics of visits and predictors of admission from a paediatric emergency room in Saudi Arabia

**DOI:** 10.1186/s12873-021-00492-6

**Published:** 2021-08-29

**Authors:** Mohammad H. Al-Qahtani, Abdullah A. Yousef, Bassam H. Awary, Waleed H. Albuali, Mohammed A. Al Ghamdi, Reem S. AlOmar, Nouf A. AlShamlan, Haneen A. Yousef, Sameerah Motabgani, Naheel A. AlAmer, Kawthar M. Alsawad, Fatimah Y. Altaweel, Kawther S. Altaweel, Roaya A. AlQunais, Fatima A. Alsubaie, Malak A. Al Shammari

**Affiliations:** 1 Department of Paediatrics, Imam Abdulrahman Bin Faisal University, College of Medicine, Dammam, Saudi Arabia; 2 King Fahd Hospital of the University, Al-Khobar, Saudi Arabia; 3 Department of Family and Community Medicine, Imam Abdulrahman Bin University, Dammam, Saudi Arabia; 4 College of Medicine, Imam Abdulrahman bin Faisal University, Dammam, Saudi Arabia

**Correction to: BMC Emerg Med 21, 72 (2021)**

**https://doi.org/10.1186/s12873-021-00467-7**

The original article [1] contained minor typos in the Abstract and References which have since been corrected.
